# Emotional Expression on Social Media Support Forums for Substance Cessation: Observational Study of Text-Based Reddit Posts

**DOI:** 10.2196/45267

**Published:** 2023-07-19

**Authors:** Genevieve Yang, Sarah G King, Hung-Mo Lin, Rita Z Goldstein

**Affiliations:** 1 Department of Psychiatry Icahn School of Medicine at Mount Sinai New York City, NY United States; 2 Department of Neuroscience Icahn School of Medicine at Mount Sinai New York City, NY United States; 3 Department of Anesthesiology Yale School of Medicine Yale University New Haven, CT United States; 4 Yale Center for Analytical Sciences, Yale School of Public Health Yale University New Haven, CT United States

**Keywords:** sentiment analysis, text mining, addiction phenotype, subjective experience phenotype, naturalistic big data, natural language processing, phenomenology, experience sampling

## Abstract

**Background:**

Substance use disorder is characterized by distinct cognitive processes involved in emotion regulation as well as unique emotional experiences related to the relapsing cycle of drug use and recovery. Web-based communities and the posts they generate represent an unprecedented resource for studying subjective emotional experiences, capturing population types and sizes not typically available in the laboratory. Here, we mined text data from Reddit, a social media website that hosts discussions from pseudonymous users on specific topic forums, including forums for individuals who are trying to abstain from using drugs, to explore the putative specificity of the emotional experience of substance cessation.

**Objective:**

An important motivation for this study was to investigate transdiagnostic clues that could ultimately be used for mental health outreach. Specifically, we aimed to characterize the emotions associated with cessation of 3 major substances and compare them to emotional experiences reported in nonsubstance cessation posts, including on forums related to psychiatric conditions of high comorbidity with addiction.

**Methods:**

Raw text from 2 million posts made, respectively, in the fall of 2020 (discovery data set) and fall of 2019 (replication data set) were obtained from 394 forums hosted by Reddit through the application programming interface. We quantified emotion word frequencies in 3 substance cessation forums for alcohol, nicotine, and cannabis topic categories and performed comparisons with general forums. Emotion word frequencies were classified into distinct categories and represented as a multidimensional emotion vector for each forum. We further quantified the degree of emotional resemblance between different forums by computing cosine similarity on these vectorized representations. For substance cessation posts with self-reported time since last use, we explored changes in the use of emotion words as a function of abstinence duration.

**Results:**

Compared to posts from general forums, substance cessation posts showed more expressions of anxiety, disgust, pride, and gratitude words. “Anxiety” emotion words were attenuated for abstinence durations >100 days compared to shorter durations (*t*_12_=3.08, 2-tailed; *P*=.001). The cosine similarity analysis identified an emotion profile preferentially expressed in the cessation posts across substances, with lesser but still prominent similarities to posts about social anxiety and attention-deficit/hyperactivity disorder. These results were replicated in the 2019 (pre–COVID-19) data and were distinct from control analyses using nonemotion words.

**Conclusions:**

We identified a unique subjective experience phenotype of emotions associated with the cessation of 3 major substances, replicable across 2 time periods, with changes as a function of abstinence duration. Although to a lesser extent, this phenotype also quantifiably resembled the emotion phenomenology of other relevant subjective experiences (social anxiety and attention-deficit/hyperactivity disorder). Taken together, these transdiagnostic results suggest a novel approach for the future identification of at-risk populations, allowing for the development and deployment of specific and timely interventions.

## Introduction

### Background

Substance use disorders afflict millions of Americans, exacting an immeasurable toll on individuals and communities. A core feature of drug addiction is the emergence of withdrawal symptoms characterized by negative affective states that, through negative reinforcement, contribute to a relapsing cycle of continued drug use despite adverse consequences [1,2]. Emerging evidence suggests that subjective emotional experiences while using a drug [3,4], and during abstinence [5,6], may be a powerful predictor of problem drug use behavior both for that drug and, notably, other substances as well [7], underscoring the significance of emotion regulation and expression to substance use disorders in general. An important challenge in the characterization and treatment of drug addiction is to identify the role-specific emotions play in the escalation of substance use and in its cessation.

Understanding the subjective experiences of substance use and cessation might also provide valuable insights into the epidemiological links between addiction and related disorders. When compared with the general population, problem drug use and addiction are highly prevalent in clinical populations with nonsubstance-related mental health disorders that encompass impairments in emotion regulation, especially mood disorders (including depression), anxiety, psychotic disorders, and attention-deficit/hyperactivity disorder (ADHD) [8]. The mechanisms underlying such comorbidities with substance use disorders are poorly understood, although they likely reflect clusters of shared neurobiological predispositions and environmental risk factors [9,10]. Individuals with comorbid substance and nonsubstance disorders frequently experience poorer abstinence-based treatment outcomes [8], highlighting the therapeutic importance of transdiagnostic approaches to studying drug addiction.

### Prior Work

A key element of subjective experiences is emotional content, which can be extracted from verbal reports such as in speech or text. Importantly, social media has become a ubiquitous channel for text-based public discourse on topics including mental illness and drug use or addiction. A rapidly increasing number of studies are now tapping into publicly accessible, user-generated, and user-friendly social media content and databases, which facilitate the gathering of large amounts of targeted data, capturing population types and sample sizes that are not typically amenable to laboratory-based studies. Such studies have been applied to understanding current and epidemiological trends in public health [11], such as attitudes toward COVID-19 [12,13] and substance use disorders [14], exposing patterns of human behavior that may not be otherwise captured through standard experimental and clinical tools. As a result, web-based discussion forums such as the social media platform Reddit have become a vital resource for the large-scale collection of ever-expanding naturalistic, and topical, health data.

Such data sets are highly suitable for natural language processing (NLP) analysis methods, providing ubiquitous and readily accessible measures of human behavior that can be mined for public opinion and marketing purposes, but also public health research. On Reddit, pseudonymous users submit posts and post replies to single-topic discussion forums called subreddits (see Table 1 for an example post), including a variety of mental health subreddits, with some that offer support for substance use cessation. Using a variety of NLP-based analyses, studies using data from Reddit (as well as Twitter, Facebook, and Instagram) have effectively applied a naturalistic lens to how individuals communicate about drugs, including their own consumption patterns and attempts to quit or abstain [15-17]. A potentially powerful application of these studies is the ability to predict future drug use, both at the population (ie, epidemiological trends in specific drug classes) and individual (ie, the propensity to relapse) levels. Considering the already established role that subjective emotional experiences play in addiction vulnerability and problem drug use, the emotional content of spontaneously generated self-reports might provide an especially valuable predictor of an individual’s future drug use and markers of recovery, which have been relatively neglected in the current addiction literature. Importantly, anonymous social media data can capture users’ candid experiences with substances and substance use or cessation in real time, including individual emotional accounts associated with success or failure to abstain.

**Table 1:** Example posts from substance cessation subreddits.

Subreddit	Title	Text
r/stopdrinking	I just realized that Thanksgiving will be exactly 60 days sober for me	A year ago I couldn’t even imagine a life free from alcohol. I have so much to be thankful for even with all the nonsense going on these days.
r/stopsmoking	Here we go again	My fourth time seriously trying to quit! I’m at 4 days 8 hours right now and I am very proud of myself. It hasn’t been easy though. Anyone else have experience with…
r/leaves	5 months 23 days	I never thought I’d make it this far but I’m doing great. I’m feeling in control, I don’t need to smoke any time I get bored or sad or lonely or stressed, I have money to spend…

### Objective

Using 2 million Reddit posts each from the fall of 2020 and the fall of 2019 across 394 topic categories, we studied emotional content in posts from 3 major substance cessation forums (alcohol, nicotine, and cannabis) as compared to nonsubstance cessation posts, with the following objectives: (1) to identify unique patterns and specificity by comparing results to the largest nonsubstance cessation subreddits (those exceeding 1 million members), allowing for transdiagnostic comparisons; (2) to identify general patterns by exploring cross-substance similarities in emotions expressed in the 3 substance cessation forums; (3) to characterize changes in patterns of emotion expression as a function of self-reported abstinence; and (4) to examine the reproducibility of findings by comparing 2 data sets (a post–COVID-19 discovery data set and a pre–COVID-19 replication data set).

## Methods

### Feature Selection

We selected emotion words of interest a priori and independent of the Reddit data. To ensure reasonable coverage of common emotion categories, we used multiple resources, including the primary and secondary emotions proposed by Plutchik [18]. In total, 21 emotion categories were selected for the curated emotion word bank (Table S1 in Multimedia Appendix 1 [18-23]), and 15 categories for a control analysis using the curated time word bank (Table S2 in Multimedia Appendix 1). Using the *parsing.preprocessing* package from the Gensim open-source Python library created by Řehůřek and Sojka [24], raw text from Reddit posts were preprocessed to convert all letters to lowercase and strip all punctuation, multiple white spaces, and HTML tags (eg, tags for bolding or italicization).

### Search Strategy and Selection Criteria

The exemplar substance cessation subreddits for the 3 most commonly used drug classes were identified by using internet searches for “Reddit,” “addiction,” and the substance of interest, then choosing the subreddit result with the largest membership base. The r/stopdrinking subreddit had 256,000 members, r/stopsmoking 113,000 members, and r/leaves 153,000 members as of November 2020. In addition, we included a total of 391 comparison subreddits, each with greater than 1 million members, to capture a subject pool that might resemble the general population for a control sample, with the 3 selected substance cessation subreddits. Thus, the discovery data set comprised a total of 394 subreddits and nearly 2 million unique posts. For each subreddit, the pushshift.io Reddit application programming interface was used to extract 5025 consecutive posts (see Table 1 for an example) from late November 2020 through early January 2021.

Subreddits in which fewer than 3000 of the 5025 posts contained body text were excluded. Implementing this arbitrary cutoff (representing a decision to use as many viable posts as possible), 105 (of 394) subreddits remained (in addition to the 3 exemplar substance cessation subreddits). The subreddit with the fewest text posts passing this cutoff contained 3003 text posts (out of the original 5025). Therefore, to maintain a consistent number of posts across subreddits, only the most recent 3003 textual posts from each sample were used for analysis. We estimated that approximately 2400 unique users per subreddit generated these posts (see “Estimation of Number of Unique Subjects” in Multimedia Appendix 1 for details). The replication data set of pre-COVID-19 posts included a starting sample of the same 105 comparison subreddits from November 2019, which underwent identical quality control for textual content, resulting in a sample of 92 (13 subreddits were excluded due to low text content; Figure S1 in Multimedia Appendix 1).

For each subreddit’s 3003 posts, we quantified the number of emotion word bank matches to produce an emotion score (Figure S2 in Multimedia Appendix 1). Using a median split, we then excluded emotion-poor subreddits (those below the median). All substance cessation support subreddits in the study met the criteria for a “high emotion” classification (above the median split) when evaluated together with the 105 comparison subreddits, forming a final sample of 54 subreddits for emotion analysis (47 in the replication sample; Figure S1 in Multimedia Appendix 1).

To assess the potential effects of abstinence duration, we extracted an abstinence-tagged data set including a subset of posts from r/stopdrinking and r/stopsmoking, which allow users to self-report the number of days since their last substance use (the r/leaves subreddit does not offer this feature). First, the most recent 15,025 consecutive posts as of late November 2020 were extracted from each of these 2 subreddits and checked for the presence of metadata for abstinence duration at the time of the post. After excluding image-only posts (as described above) and posts lacking abstinence metadata, 6595 posts remained for r/stopdrinking and 1205 for r/stopsmoking. To maintain the same number of posts across subreddits, only the most recent 1205 posts from the sample of 6595 were used for r/stopdrinking.

### Choice of Primary Measure and Statistical Approach

Our primary measures were occurrence frequency and cosine similarity. For the emotion analysis, occurrence frequency was a count of the number of times words appear in each emotion category, normalized by the total number of emotion word matches, producing a percentage value for each of the 21 emotion categories (see “Emotion Word Bank” in Multimedia Appendix 1 for details). This 21-item list of percentages was computed for each subreddit independently of other subreddits. Subreddits were considered outliers on a given emotion if their word frequency fell in the 95th percentile on that emotion compared to the control sample of large subreddits of at least 1 million members. We similarly used occurrence frequency and time word categories for the time word bank. To quantify the similarity between a given pair of subreddits, we transformed the 21-item lists of emotion occurrence frequencies into 21-dimensional vectors for each subreddit and computed the cosine of the angle (cosine similarity) between each pair of vectors, corresponding to a pair of subreddits (see “Cosine Similarity” in Multimedia Appendix 1 for details). Similar procedures were used for the time word vectors (see “Time Word Bank” in Multimedia Appendix 1 for details).

**Figure 1:**
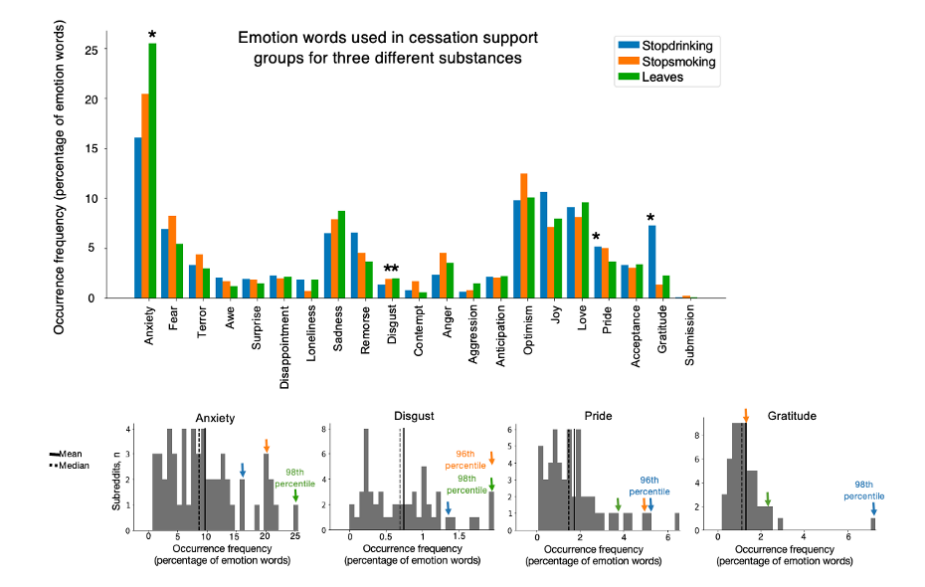
Emotion word analysis of posts from cessation support subreddits and control subreddits. The substance cessation support subreddits were compared with high-membership general interest subreddits with emotion-rich text (see Figure S1 in Multimedia Appendix 1). Top plot represents occurrence frequency of each emotion word category. Histograms show the 4 emotion words on which the substance cessation subreddits demonstrated outlier properties. Colored arrows indicate locations of the substance cessation subreddits within the larger distribution of high-emotion subreddits. Blue = r/stopdrinking, orange = r/stopsmoking, green = r/leaves. *denotes that the subreddit was an outlier (>95th percentile) in use of the target emotion words compared to control general topic subreddits.

To examine the effects of emotion (using all 21 categories) on word occurrence frequency as a function of abstinence duration, we used a 2-way ANOVA incorporating 2 independent categorical variables: emotion and abstinence duration. Due to limited post data for longer abstinence durations, we applied a cutoff of 1000 days for this analysis, with posts consolidated into 15 time bins. The abstinence factor was then divided into 2 levels denoting short-term (<100 days, 9 time bins) and long-term (>100 days, 6 time bins) abstinence. Significant interactions between emotion and abstinence duration were followed up with post hoc *t* tests. To limit multiple comparisons, we restricted the post hoc 2-sided independent samples *t* tests (comparing posts tagged with self-reported long-term abstinence vs posts tagged with short-term abstinence) to the 4 emotions identified in Figure 1, with Bonferroni correction (α=.05/4 or *P*<.01).

### Ethical Considerations

Users of Reddit’s web-based forums are made aware that their posts are publicly accessible in the website’s terms and conditions. All data were collected through the free and open-access application processing interface, which does not provide personal identifying information such as legal names, locations, or IP addresses. For the purposes of this study, we did not participate in discussions on the forums, and there was therefore no ethical consideration to inform users that their posts may be used for research. For these reasons, this study received an exemption status from Mount Sinai’s Institutional Review Board.

## Results

### Emotional Content in Substance Cessation Subreddits

The emotion compositions of the 3 substance use cessation subreddits (r/stopdrinking, r/stopsmoking, and r/leaves) similarly scored >15% (top 8/54) for “anxiety” and around 10% (top 5/54) for “sadness,” “optimism,” “joy,” and “love” (Figure 1). Furthermore, on 4 of the emotion categories, “anxiety,” “disgust,” “pride,” and “gratitude,” the substance cessation subreddits showed outlier levels of word occurrence frequency (in the 95th percentile, 3/54) compared to the full sample of subreddits (Figure 1). Specifically, the nicotine cessation subreddit (r/stopsmoking) showed high “disgust,” the cannabis cessation subreddit (r/leaves) showed a high representation for both “disgust” and “anxiety,” and the alcohol cessation subreddit (r/stopdrinking) showed a high representation for “pride” and “gratitude.” Although r/stopsmoking was also high in the “gratitude” emotion, this trend did not exceed the 95th percentile.

**Figure 2:**
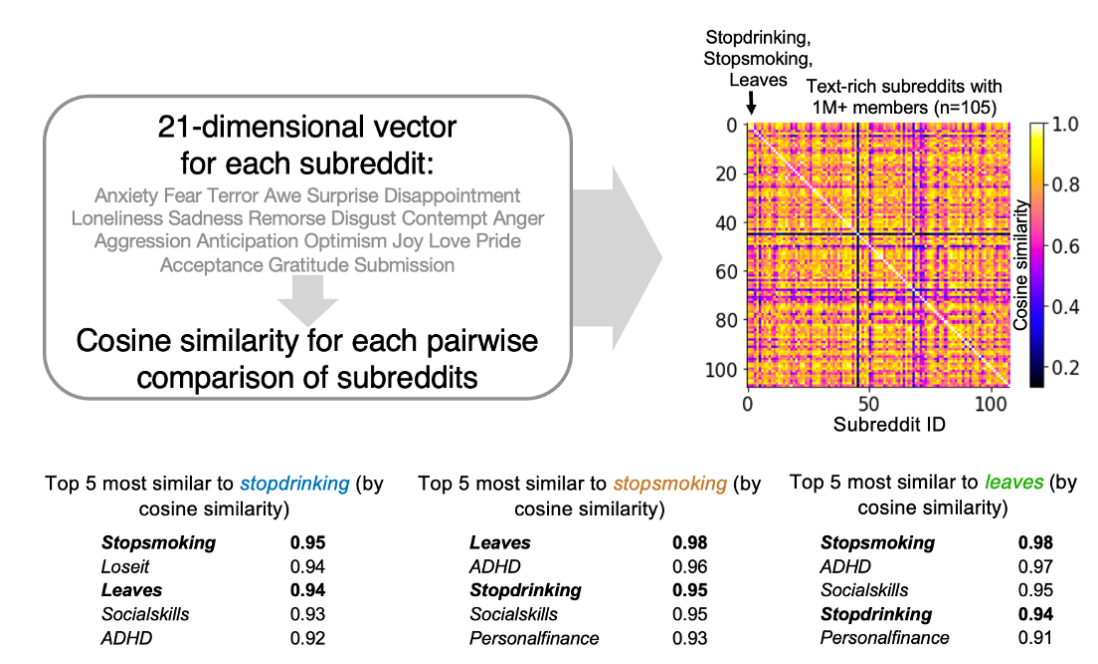
Emotional similarity of substance cessation and control subreddits. Emotion word occurrence frequencies were computed and expressed as a 21-dimensional emotion vector for each subreddit. Gray words list the emotion dimensions. Cosine similarity was computed between each pair of subreddits with respect to their emotion vectors. Heatmap illustrates the cosine similarity for pair-wise comparison analysis among all subreddits. The exemplar subreddits are at the top left (in order: r/stopdrinking, then r/stopsmoking, then r/leaves), followed by the 105 comparison subreddits in order of decreasing member size, starting with the largest: r/Showerthoughts, with over 22 million members (note: the final analysis included only the 54 most emotion dense subreddits). For each of the substance cessation subreddits, the top 5 most emotionally similar subreddits are shown with accompanying cosine similarity scores (bottom). ADHD: attention-deficit/hyperactivity disorder.

Cosine similarity analyses were performed by comparing the emotion profiles for every possible subreddit pair in our data set (Figure 2). Notably, the substance use cessation subreddits appeared consistently in the list of top 5 most emotionally similar subreddits for each of the other substance use cessation subreddits (Figure 2). Indeed, for each of these subreddits, the next most emotionally similar subreddit was one of the other substance use cessation subreddits. Additional subreddits appearing in the top 5 lists for all 3 substance cessation subreddits were r/ADHD and r/socialskills. Additionally, r/loseit, a weight-loss support subreddit, appeared in the top 5 for r/stopdrinking, and r/personalfinance, a subreddit for financial advice, appeared for both r/stopsmoking and r/leaves.

### Control Analyses: Time Words in Substance Cessation Subreddits

Cosine similarity analyses were performed by comparing the time word profiles for every possible subreddit pair in our data set similar to the emotion word analysis. Notably, the emotional similarity between the substance use cessation subreddits did not replicate for time words. Specifically, the most similar subreddits to both r/stopdrinking and r/leaves were not substance cessation subreddits (r/leaves remained the most similar to r/stopdrinking), while r/stopdrinking and r/leaves appeared in each other’s list of top 5 most similar subreddits, r/stopsmoking did not appear in either of the other 2 subreddits’ lists or vice versa (see Figure S2 in Multimedia Appendix 1). Interestingly, in these time word analyses, only 1 substance use cessation subreddit displayed outlier properties: r/stopsmoking for “morning” words (see Figure S3 in Multimedia Appendix 1).

### Emotional Content and Abstinence

**Figure 3:**
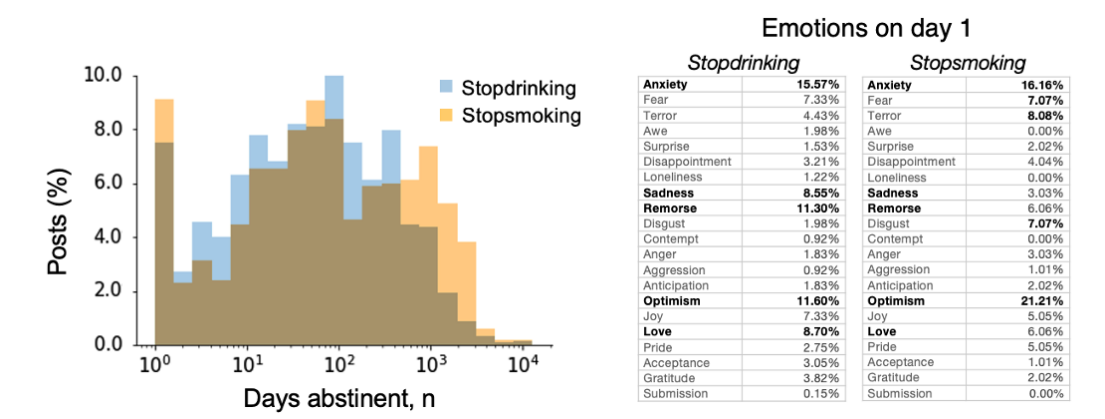
Posts on substance cessation subreddits in the first 24 hours of abstinence expressing anxiety and optimism. Analysis of post metadata on self-reported abstinence from 2 substance cessation subreddits, r/stopdrinking and r/stopsmoking. Left plot shows frequencies of posts by length of abstinence. The x-axis is logarithmic and extends to 10,000 days. The table shows word occurrence frequencies in each emotion category restricted to abstinence-tagged posts made within the first 24 hours of abstinence. The top 5 most prevalent emotion word categories are in bold format. Percentage scores express the occurrence frequency of words from the target emotion category relative to the total frequency of any word match for the emotion word bank.

Posting patterns were assessed for subreddits with self-reported abstinence duration metadata (r/stopdrinking and r/stopsmoking; Figure 3). Both subreddits showed a disproportionately high post volume in the first 24 hours of abstinence. That is, in both subreddits, at least 7% (462/1205) of all posts were made within the first 24 hours of abstinence, which is more than 2 orders of magnitude greater than chance expectations (ie, given that the abstinence period range extended to over 10,000 days for each subreddit, if users were equally likely to post at any abstinence duration, only 0.01%, n=1, of posts would be expected to be made within the first 24 hours). In addition, for both subreddits, posting tendencies increased around the 100th day of abstinence.

Given the high frequency of posting within the first 24 hours of abstinence, we quantified the emotion profiles of posts during this period (Figure 3). For both r/stopdrinking and r/stopsmoking, the 2 most highly represented emotions on the first day of abstinence were “anxiety” and “optimism.” The next 3 most highly represented emotions were different for the 2 subreddits: “remorse,” “love,” and “sadness” for r/stopdrinking and “terror,” “disgust,” and “fear” for r/stopsmoking.

**Figure 4:**
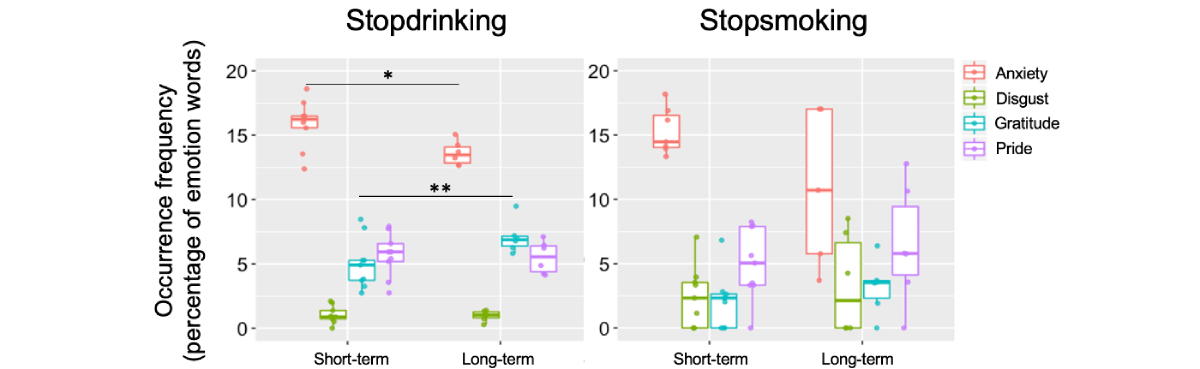
Emotions expressed during the first 1000 days of abstinence. Analysis of post metadata on self-reported abstinence from 2 substance cessation subreddits. Occurrence frequency is shown for emotion words from the 4 emotion categories identified in Figure 1 for short-term (<100 days) and long-term (>100 days) abstinence time points. Long-term abstinence showed a decrease in “anxiety” and a trend toward an increase in “gratitude” emotion expression as compared with the short-term time points for r/stopdrinking. The legend applies to both plots. *significant at *P*<.05, Bonferroni corrected, **approached significance, did not survive Bonferroni correction.

Importantly, ANOVA results examining emotion category and abstinence duration relationships for the r/stopdrinking data showed a main effect of emotion category (*F*_20,1_=156.8; *P*<.001), no main effect of abstinence duration (*F*_20,1_=0.00; *P*=1.00), and a significant interaction between emotion category and abstinence duration (interaction effect *F*_20,1_=3.80; *P*<.001), suggesting a shift in emotion word expression at longer compared to shorter abstinence durations (Figure 4). Post hoc *t* tests on the r/stopdrinking data restricted to the original 4 emotions of interest identified in Figure 1 (“anxiety,” “disgust,” “pride,” and “gratitude”) confirmed that the “anxiety” emotion was expressed significantly less for abstinence durations greater than 100 days compared to shorter durations (*t*_12_=3.08, 2-tailed; *P*=.01). The r/stopdrinking data also showed higher “gratitude” emotion expression for longer abstinence durations (greater than 100 days), but this effect did not survive Bonferroni correction for multiple comparisons (*t*_13_=−2.44, 2-tailed; *P*=.03). The ANOVA results for the r/stopsmoking data showed a main effect of emotion (*F*_20,1_=20.0; *P*<.001), no main effect of abstinence duration (*F*_20,1_=0.00; *P*=.99), and no effect of the interaction between emotion and abstinence duration (*F*_20,1_=1.45; *P*=.09).

### Replication Analysis

**Figure 5:**
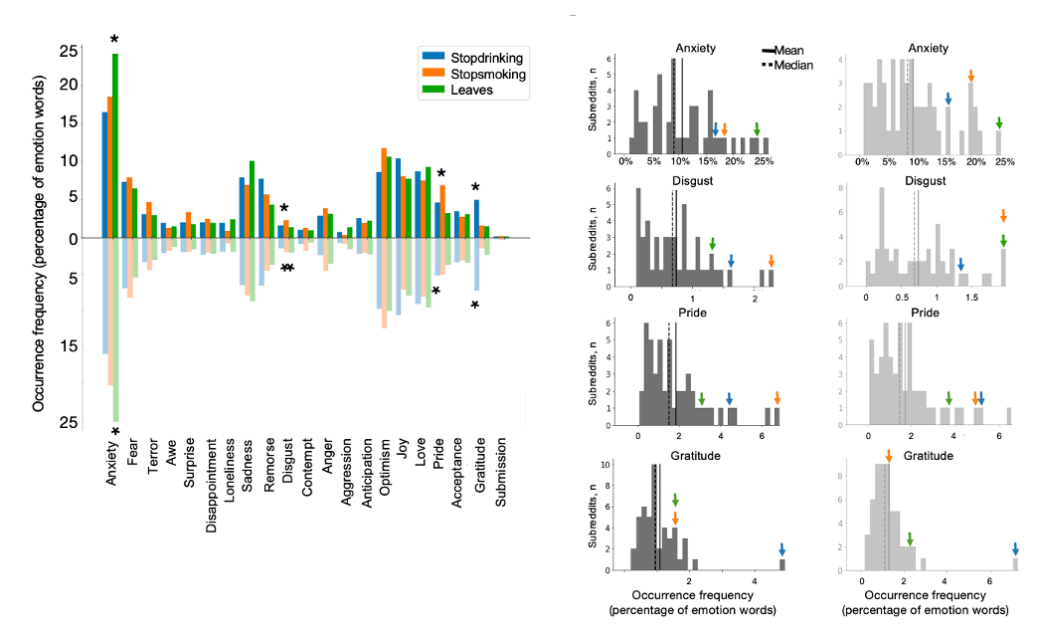
Emotion word analysis in the replication data set. Left plot represents emotion word occurrence frequencies for each exemplar substance cessation subreddit, using posts from the replication data set. Occurrence frequencies are expressed as a percentage of all emotion words counted in that subreddit’s posts. The corresponding graph from the discovery data set (Figure 1) is reproduced below the replication data set results as a translucent “reflection” for ease of visual comparison. Histograms show the 4 emotion categories on which the substance cessation subreddits demonstrated outlier properties. Colored arrows indicate locations of the substance cessation subreddits within the larger distribution of high emotion subreddits. Blue = r/stopdrinking, orange = r/stopsmoking, green = r/leaves. *denotes that the subreddit was an outlier (>95th percentile) in use of the target emotion words compared to general topic subreddits.

Results from the replication data set were compared with the discovery data set (Figure 5). Similar to the previous results, the same 4 outlier emotions were identified in the replication data set. At least one of the substance cessation subreddits showed outlier representation in the top 95th percentile (2/47) for “anxiety,” “disgust,” “pride,” and “gratitude” emotion categories relative to comparison subreddits. Specifically, the outlier status remained robust for r/leaves on “anxiety,” r/stopsmoking on “disgust,” and r/stopdrinking on “gratitude.” The emotion word cosine similarity showed similar results in this replication data set compared to the discovery data set (see Figure S4 in Multimedia Appendix 1).

## Discussion

### Principal Results

Here, we used spontaneous, self-directed reports provided under relative anonymity in a naturalistic setting (via public forums available on Reddit) generated by hundreds of thousands of subjects to examine the phenomenology of the subjective experience of substance use cessation across 3 of the most common substances of abuse. To apply a transdiagnostic lens, we chose emotion words instead of more addiction-specific features, allowing us to contrast results against the control population of Reddit users. We also compared results as a function of time since last reported drug use and across 2 time periods. Our results suggest an emotional signature preferentially expressed across the alcohol, nicotine, and cannabis cessation subreddits, displaying outlier properties on 4 emotions. These results replicated across 2 time periods (pre- and post-COVID-19), demonstrating continuity in the observed effects over time despite major societal shifts imposed by the pandemic that impacted drug use in general [25]. Significant interactions with time suggested that the identified emotional signature may change with abstinence duration. Although distinct from the control population, this emotion profile showed notable similarity to a few other nonsubstance-themed populations (eg, those plausibly experiencing ADHD in r/ADHD, social anxiety in r/socialskills, financial stress in r/personalfinance, or attempting weight loss in r/loseit), suggestive of transdiagnostic clinical relevance as well as common demographic characteristics.

First, we captured 4 emotions (“disgust,” “anxiety,” “pride,” and “gratitude”) that were expressed within the 3 substance cessation subreddits at a level exceeding 95% (top 3/54) of all included subreddits. The smoking cessation subreddit’s outlier status on “disgust” is consistent with research supporting the role of disgust (as opposed to other emotions, such as health-related anxiety) in reducing craving in smokers [26-29]. Notably, cocaine is another stimulant drug of abuse that is frequently smoked (in the crack form), and cocaine-dependent individuals also show aberrant disgust responses [30]. Aberrant disgust responses are also implicated in opiate [31] and internet addiction [32]. The cannabis cessation subreddit also demonstrated outlier status on “disgust” although here it was only observed for the discovery data, suggesting that this aspect of the subjective experience of cannabis cessation may be intensifying over time. The use of “anxiety” was also high in this subreddit, consistent with well-established reports of a link between cannabis use and anxiety [33,34], particularly social anxiety [35-37]. The alcohol cessation subreddit demonstrated outlier status on the “pride” and “gratitude” emotions, the latter was consistent with the well-established role of gratitude in Alcoholics Anonymous (as well as Narcotics Anonymous) programs [19,38,39], and theories linking gratitude with improved outcomes in alcohol use disorder [40]. The smoking subreddit was also highly ranked on the “pride” emotion, although not always exceeding the 95th percentile in representation. In general, relatively less research [41] has been devoted to the role of pride in substance use cessation [19].

Using cosine similarity to quantify shared emotion patterns, specifically of relative occurrence frequencies across emotion words, we showed that the 3 substance cessation subreddits were more emotionally similar to each other than to other subreddits in the sample. Nevertheless, this substance cessation emotion phenotype appeared, to a lesser degree, in other relevant subreddits, for example, those concerned with social anxiety and ADHD. Consistent with this finding, young adults with ADHD are 1.5 times more likely to develop a substance use disorder for alcohol, cigarette smoking, and illicit drugs (for which cannabis is the most common drug of abuse) [42]. One question raised by this phenomenological overlap in emotion profiles is whether the implicated disorders (eg, substance use disorder but also social anxiety and ADHD) share underlying predisposing conditions or genetic factors that affect multiple emotion system traits [43]. This finding may also have important implications for research into potential shared neurobiological correlates as well as policy implications for targeting public health prevention and intervention efforts.

Our abstinence-linked analyses of r/stopdrinking and r/stopsmoking showed a disproportionately high tendency to post within the first 24 hours of abstinence. This finding suggests that, across substances, these first 24 hours may be a special window associated with the greatest inclination to engage with support communities, possibly offering a key intervention point in recovery from addiction. This result contrasts with the culture of some offline support groups, for instance, Cocaine Anonymous, which discourages members from sharing at meetings within the first 24 hours of abstinence [44]. Beyond the first day, a significant drop in the “anxiety” emotion was observed for longer compared to shorter durations of abstinence from alcohol, consistent with prior reports suggesting that anxiety linked to alcohol cessation tends to resolve after about 6 weeks of abstinence [45]. Although not significant following Bonferroni correction, we speculate that the finding of greater “gratitude” emotion with longer alcohol abstinence duration may suggest a cumulative effect of exposure to alcohol cessation support groups, which are strongly influenced by the culture of gratitude at Alcoholics Anonymous. While these results await replication with objectively verified abstinence duration (eg, by using urine drug screens and prospective longitudinal designs), these changes in emotion expression may also be linked to the time course of craving incubation [46,47].

The 4 outlier emotions identified above, as well as the overall substance-preferential emotion pattern identified in our cosine similarity analysis, were robustly replicated across 2 different time periods, pre- and post–COVID-19, during the same season of the year (late autumn to early winter). The transdiagnostic connections to nonsubstance-themed subreddits were also robust, with r/ADHD and r/socialskills surfacing in both pre- and post-COVID-19 results. This degree of replication was especially powerful given that the COVID-19 pandemic was previously shown to have affected posting behavior on Reddit, particularly within support groups, including spikes in posts relating to health anxiety across multiple forums [48]. The reproducibility of these emotion profile properties is therefore suggestive of a stable subjective experience phenotype related to substance cessation.

### Limitations

We first acknowledge the absence of independent or objective measures as a limitation to validating these subjective reports. We note that the characteristics of the data set do not permit verification of a clinical diagnosis. Therefore, the degree to which this substance cessation-preferential emotion profile may explain individual variation in disease risk, progression, prognosis (eg, relapse risk), or phase in abstinence remains unknown. Second, our data set is limited by selection effects that bias our findings toward subjects who are English literate, have access to the internet, and are motivated to self-disclose in written form about these potentially sensitive topics. In general, it is important to acknowledge potential bias introduced by the overall demographics of Reddit users, who are predominantly White, male, and college-educated individuals who are 18-29 years of age and living in the United States [49]. Other selection biases include geographic and temporal constraints in the collected data, which may also influence sentiment expression [50,51]. These biases may have excluded many other groups of individuals struggling with substance cessation, and the overlap with participants in laboratory-based studies may be limited. It is also important to note that the substance cessation subreddits were smaller (less than 1 million members) than the subreddits in the comparison sample (all with at least 1 million members), which represented the “general population” control sample. This difference in membership size is an unavoidable potential to confound in comparing control population interests with a relatively niche interest. However, given that the preferential similarity across the substance cessation subreddits did not replicate when using a time word bank, our results argue against a trivial consequence of a difference in membership size. We also cannot exclude the possibility that some emotion similarities identified arose from overlapping membership across substance cessation subreddits and other subreddits such as r/ADHD, although a recent subreddit similarity analysis based only on shared membership identified relationships distinct from our results [52]. Future efforts could use pretrained language models [53] or transformer models [54,55], which could capture context differently than single words. For example, automated tools for NLP can leverage a wider range of semantic and lexical features in speech and text, such as emotional valence and negation of emotion words [56], pronoun use [57], and linguistic complexity [58], which may provide a richer selection of features for the sentiment analysis. While not directly pertinent for the purposes of this study, which intended to identify the frequency of commonly expressed emotions on substance cessation forums, these concepts could add a level of specificity to the interpretation of the results. Future analyses may also benefit from sampling more deeply on the order of tens of thousands or hundreds of thousands of posts. Nevertheless, our use of a priori selected word banks to identify specific patterns of emotional expression suggests that important emotional features can be captured in these data even at the relatively small scale of 5000 raw posts extracted per subreddit.

### Conclusions

In conclusion, our results identify a reproducible subjective experience phenotype linked to a key phase of recovery in addiction: substance cessation. This phenotype showed extensive cross-substance overlap, was emotion specific, and may be partly generalizable to other related nonsubstance mental health conditions. Our approach and results could open a window into assessment and prevention efforts in at-risk substance-naive subjects. For example, our methods are conducive to targeting large populations with a strong presence on web-based social media (eg, adolescents and young adults) as well as other at-risk groups, allowing for reach to those not commonly enrolling in research or treatment. Substance use cessation prevention messages (including a link to available resources) may thus be included in sidebars for the r/ADHD subreddit, and anxiety support resources might be introduced into any of the substance cessation subreddits. Further, results can be used to better target the wording of public service announcements (including those used by social media and related technology), tailored to key time windows within the substance cessation trajectory. For example, interventions addressing anxious feelings may be best targeted at recently abstinent populations, whereas gratitude-laden messaging might be more appropriate for longer-term abstainers. In general, our results support ongoing efforts to characterize potential unique features of language use in populations with addiction, explored using NLP to identify behaviors that characterize substance use disorders or predict longitudinal drug use outcomes. If validated in a population clinically diagnosed with addiction, these results can inform timely prevention and intervention efforts on a large scale.

## Appendix

Multimedia Appendix 1 Supplementary information supporting methods and results in the main text.

## Abbreviations

ADHD: attention-deficit/hyperactivity disorder
NLP: natural language processing

## Data Availability

The data sets analyzed during this study are available in Open Science Framework Storage: DOI 10.17605/OSF.IO/FCG8V.
