# Supplementary material for: Emotional Expression on Social Media Support Forums for Substance Cessation: Observational Study of Text-Based Reddit Posts
Source: J Med Internet Res. 2023 Jul 19;25:e45267. doi: 10.2196/45267 (PMC10398365; doi:10.2196/45267)
Supplement: Multimedia Appendix 1 [file jmir_v25i1e45267_app1.docx]

**Multimedia Appendix 1**

**S1. Estimation of Number of Unique Subjects**

When estimating the number of unique participants represented in our data, we consider that: (i) users may create multiple usernames, and (ii) some users may delete their accounts, resulting in several posts where the username is displayed as “[deleted].” The former issue is an inherent limitation considering user pseudonymity on Reddit. To address the latter, we used the following method for estimating the number of unique users involved in the study: for each subreddit sample of 3,003 posts (obtained after applying quality control), we first quantified the number of unique usernames, excluding the special name “[deleted],” as well as the number of posts attributed to “[deleted].” For example, the r/stopdrinking sample had 2,129 unique usernames excluding “[deleted].” Fifty-seven posts from the r/stopdrinking sample were attributed to “[deleted],” leaving 2,946 (3,003 – 57) posts attributed to users other than “[deleted]” or approximately 1.38 posts per user (2,946/2,129 unique usernames). Applying this posts-per-user estimate, the 57 posts by “[deleted]” are estimated to belong to 57/1.38 or 41 unique users. The final upper bound estimate for total unique users contributing to the r/stopdrinking sample of posts would therefore be 2,170 users (2129 + 41). Using the above approach, we estimate that our discovery dataset of posts from r/stopdrinking, r/stopsmoking, and r/leaves comprises 2,170, 2,357, and 2,139 subjects, respectively. Examining comparison subreddit samples of 3,003 posts each from the discovery dataset, the estimated number of unique participants ranged from 1,251 to 2,810, with a median participant pool of 2,390 (mean 2,348).

**Emotion Word Bank**

For the purposes of our analysis, we considered employing an openly available lexicon for emotion and sentiment analysis. However, considering the specific characteristics of our population of interest, we determined that general tools were not suitable given the absence of certain emotion categories that are pertinent to individuals trying to abstain from drugs, as well as the presence of terms with additional non-emotion meanings that have unique meaning to this population. For these reasons, we decided to curate a custom lexicon *a priori* that includes emotion categories and emotion words that are better targeted to our purposes. As a starting point, we chose the primary and secondary emotions proposed by Plutchik [18]: fear, awe, surprise, disappointment, sadness, remorse, disgust, contempt, anger, aggressiveness, anticipation, optimism, joy, love, acceptance, and submission, due to the general acceptance of these emotions as common to subjective experience, and straightforward arrangement of each emotion relative to the others. Due to the well-established role of positive psychology concepts, particularly gratitude, in 12-step program substance use cessation support groups [19], we added three more emotions: grateful, proud, and lonely. Since many words might be grouped to refer to the same emotion (e.g., anger, angry, rage), these emotions were further expanded to become emotion categories, often containing multiple similar words. Given the particularly robust representation of fear-related words within the first 5,000 most common English words [20], the “fear” category was further stratified into “anxiety,” “fear,” and “terror” categories in order to represent different levels of intensity. Next, common emotion words were collected from multiple web sources identified by Internet searches [21,22] to populate the emotion categories. A curated subset of these emotion words matching the above categories was added to the emotion word bank. Words that evoked emotion without necessarily being a word for a specific emotion were excluded; for example, “slam” was not added to the “anger” word bank. Words with potentially problematic dual meanings were also excluded, such as “bitter,” which could potentially refer to a taste (especially relevant to substance cessation/withdrawal effects) and not to an emotion. The final emotion word bank was not intended to be an exhaustive list of all possible emotions, or a list of all common misspellings, but was aimed at providing wide coverage of a spectrum of emotions.

**Time Word Bank**

To choose time-related words, we began with the *a priori* decision to designate time categories that correspond to major times of day (“morning,” “midday,” “afternoon,” and “night”) and major days or day types (“Monday,” “Tuesday,” “Wednesday,” “Thursday,” “Friday,” “Saturday,” “Sunday,” “weekend,” “weekday,” “vacation,” “holiday”). To populate these categories, we curated a selection of time-linked words, e.g., “dawn,” “lunch,” “bedtime” (see Table S2 for full list), which were sorted into the corresponding major categories. Next, to find additional semantically related words that might fall into these time categories, we used Google’s pre-trained word2vec model [23]. The model uses word vectors of 300 features each, comprising a vocabulary of three million words and phrases that were extracted from training on approximately 100 billion words from a Google News dataset. Beginning with our original time words above as “seed” words, we used the word2vec model to retrieve a list of words most similar to the “seed” word. Similarity was quantified using cosine similarity, a common metric in text analysis that measures the cosine of the angle between two nonzero feature vectors. We were not able to use all results returned in this manner, for reasons that are illustrated by this example result, obtained by searching for all words in the word2vec model with cosine similarity > 0.5 compared to “night,” displayed below:

[“night,” 0.9999999], [“evening,” 0.8094645], [“afternoon,” 0.70728827], [“nights,” 0.6702841], [“Saturday,” 0.6503213], [“morning,” 0.64529103], [“tonight,” 0.61351067], [“Sunday,” 0.59564334], [“weekend,” 0.5792553], [“Friday,” 0.5687226], [“wee_hours,” 0.5450467], [“nigth,” 0.52262586], [“Monday,” 0.5161948], [“Thursday,” 0.5159596], [“Tuesday,” 0.51013184], [“New_Year_Eve,” 0.5089484], [“Thomas_Willgruber_died,” 0.5084885], [“Wednesday,” 0.5078756], [“day,” 0.50700086], [“afteroon,” 0.50638497], [“primetime_daytime,” 0.5059618], [“midnight,” 0.5019987], [“week,” 0.5005937]

Some results in the above list are clearly ill-suited for the “night” category given a better semantic match with other categories (such as “day”). In addition, some results were not useful for invoking the notion of nighttime, such as “Thomas_Willgruber_died.” Therefore, the results from using the word2vec model were manually curated before including a subset of results in the time word bank. The common misspellings identified by the word2vec model were included in the time word bank where appropriate.

**Cosine Similarity**

The cosine similarity measure was used to quantify similarities between subreddits in terms of their representation on the emotion and time word banks. Specifically, within each word bank, categories of words (21 for the emotion word bank and 15 for the time word bank) had an accompanying occurrence frequency score, represented as a percentage. These lists of category scores were represented as n-dimensional vectors, where n is the number of percentage values. Cosine similarity between two subreddits on a given word bank was measured by computing the cosine of the angle between the two corresponding vectors.

| **Table S1. Emotion word bank, including common misspellings.** | |
| --- | --- |
| **Emotion Category** | **Words** |
| anxious | apprehension, apprehensive, anxious, anxiously, anxiety, jittery, antsy, nervous, restless, concerned, worried, fretful, uneasy |
| fear | frightened, fearful, scared, afraid, fear, skittish, jumpy |
| terror | terrified, desperate, terror, horrified, horror, horrible |
| awe | awe, reverent, reverence, wonder, inspired, inspiring |
| surprise | surprised, surprise, distracted, distraction, amazed, amazement, stunned, shocked |
| disappointment | disappointment, disappointed, disheartened, frazzled, hopeless, overwhelmed |
| lonely | lonely, dateless, loveless, desolate, friendless, rootless, loner, solitude, lonelier, forlorn, loneliest, loneliness, lonesome |
| sad | unhappy, distraught, upset, sad, melancholy, morose, sadness, pensive, grief, grieving, depressing, depressed |
| remorse | remorse, guilt, guilty, embarrassed, embarassed, embarrassment, embarassment, ashamed, shame |
| disgust | disgust, disgusted, boredom, loathe, loathing |
| contempt | contempt, impatient, frustrated, grumpy, offense, offended, offensive |
| anger | angry, rage, sullen, anger, annoyance |
| aggressive | aggressive, aggressiveness, agitated, suspicious, paranoid |
| anticipation | anticipation, eager, excited, eagerly, excitedly, vigilance, vigilant, alert, attentive |
| optimism | optimism, optimistic, hopeful, hoping, hope, confident |
| joy | happy, elated, ecstatic, jovial, joy, ecstasy, satisfied, satisfy, amused, amusing, hilarious, funny |
| love | love, adore, adoration, loving, adoring |
| proud | pride, proud |
| acceptance | acceptance, relaxed, relaxing, relaxation, laxed, easygoing, relaxes, calmer, laidback, comfortable, relax |
| grateful | grateful, gratitude, thankful |
| submissive | submissive, hesitant, reluctant |

| **Table S2. Time word bank, including common misspellings.** | |
| --- | --- |
| **Time Category** | **Words** |
| morning | dawn, morning, sunrise, daybreak, moring, midmorning, monring, morn_ing, mornings, mornng, 7am, 8am, 4a.m., 7a.m., 5a.m., pre_dawn, 7AM, 5am, 8am_AEDT, 9am, morn |
| midday | noon, lunch, midday, lunchtime, 1pm, 2pm |
| afternoon | afternoon, afteroon, afternon, midafternoon, afernoon, afternnon, afternooon, 5pm, 3pm, 4pm |
| night | sunset, nightfall, evening, night, bedtime, midnight, nights, wee_hours, tonight, nigth, overnight |
| monday | monday |
| tuesday | tuesday |
| wednesday | wednesday |
| thursday | thursday |
| friday | friday |
| saturday | saturday |
| sunday | sunday |
| weekend | weekend |
| weekday | weekday |
| vacation | vacation, vacay, staycay, staycation |
| holiday | holiday, new years eve, thanksgiving, christmas, xmas, valentines, st patrick, new years, easter, yom kippur, rosh hashanah, hanukkah, mothers day, fathers day, presidents day, MLK, veterans day, independence day, july 4, fourth of july, labor day, halloween |


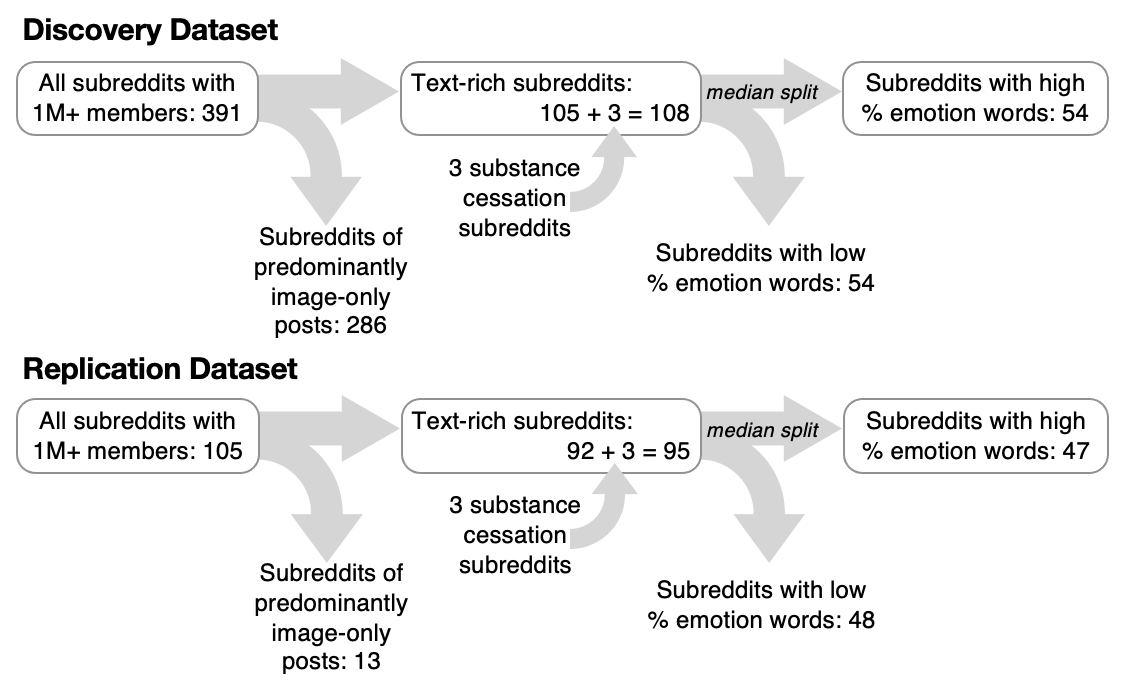


**Figure S1.** Overview of the dataflow for the discovery and replication datasets. Text-poor subreddits were excluded, as were subreddits with low prevalence of emotion words, identified by median split. The replication dataset started with the same 105 comparison subreddits that were identified as text-rich in the discovery dataset.

**
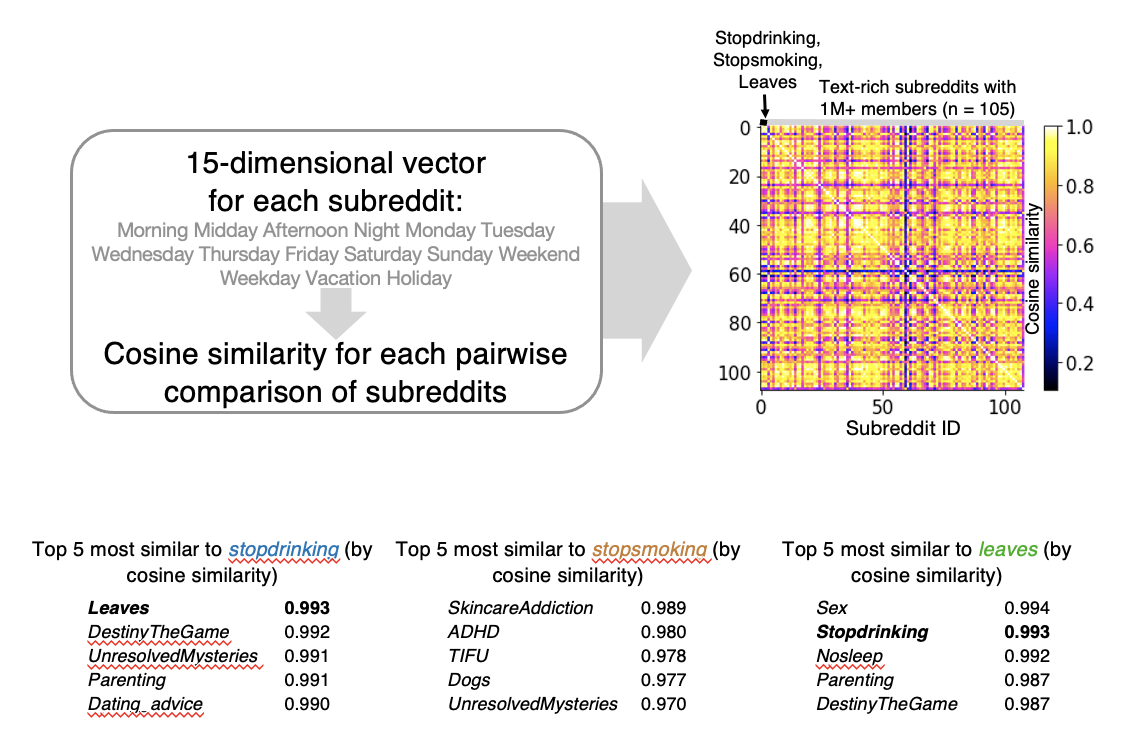
**

**Figure S2.** Time word similarity of substance cessation and control subreddits. Time word occurrence frequencies were computed and expressed as a 15-dimensional emotion vector for each subreddit used in the emotion word analysis (see Multimedia Appendix 1, Figure S1). Cosine similarity was computed between each pair of subreddits with respect to their time vectors. Heatmap illustrates the cosine similarity pair-wise comparisons analysis between all subreddits. The exemplar subreddits are at the top left (in order: r/stopdrinking, then r/stopsmoking, then r/leaves), followed by the 105 comparison subreddits in order of decreasing member size, starting with the largest: r/Showerthoughts, with over 22 million members (note: the final analysis included only the 54 most emotion dense subreddits). For each of the substance cessation subreddits, the top five most similar subreddits are shown (bottom), with accompanying cosine similarity scores.


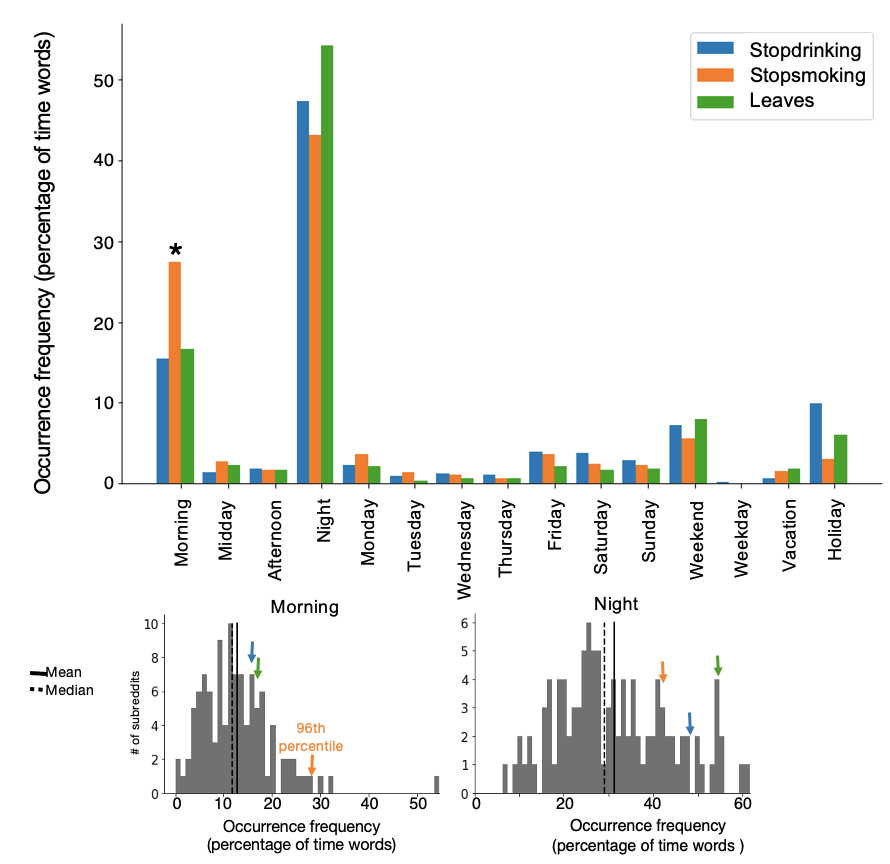


**Figure S3.** Time word analysis of posts from cessation support subreddits and control subreddits. The substance cessation support subreddits were compared with high-membership general interest subreddits with emotion-rich text (see Multimedia Appendix 1, Figure S1). Top plot represents occurrence frequency of each time word category. Histograms show the “morning” time words on which r/stopsmoking demonstrated outlier properties with “night” for comparison. Colored arrows indicate locations of the substance cessation subreddits within the larger distribution of high emotion subreddits. Blue = r/stopdrinking, orange = r/stopsmoking, green = r/leaves. *denotes that the subreddit was an outlier (>95th percentile) in use of the target emotion words, compared to “control,” general topic subreddits.


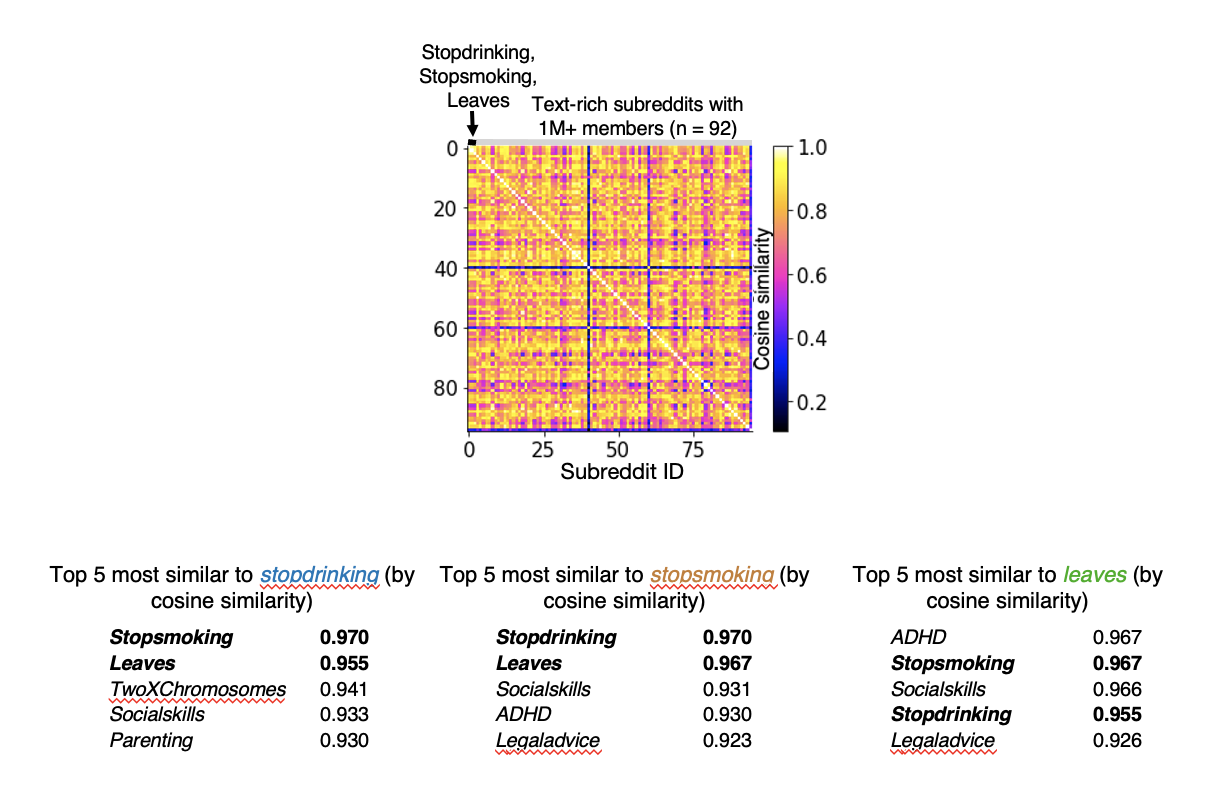


**Figure S4.** Emotion word analysis of posts from cessation support forums for three different substances compared to general forums in the replication dataset. Three substance cessation support subreddits (r/stopdrinking, r/stopsmoking, and r/leaves) were compared with high-membership general interest subreddits with emotion-rich text (see Multimedia Appendix 1, Figure S1). Heatmap illustrates the cosine similarity pair-wise comparisons analysis between all subreddits. The exemplar subreddits are at the top left (in order: r/stopdrinking, then r/stopsmoking, then r/leaves), followed by the 94 comparison subreddits in order of decreasing member size, starting with the largest: r/Showerthoughts, with over 22 million members (note: the final analysis included only the 47 most emotion dense subreddits). For each of the substance cessation subreddits, the top five most emotionally similar subreddits are shown with accompanying cosine similarity scores (bottom).
